# Assessment of Resistance and Bioremediation Ability of* Lactobacillus* Strains to Lead and Cadmium

**DOI:** 10.1155/2017/9869145

**Published:** 2017-01-04

**Authors:** Anna V. Kirillova, Anna A. Danilushkina, Denis S. Irisov, Nataliya L. Bruslik, Rawil F. Fakhrullin, Yuri A. Zakharov, Vladimir S. Bukhmin, Dina R. Yarullina

**Affiliations:** ^1^Department of Microbiology, Kazan Federal University, Kremlevskaya St. 18, Kazan 420008, Russia; ^2^Bionanotechnology Lab, Institute of Fundamental Medicine and Biology, Kazan Federal University, Kreml uramı 18, Kazan, Tatarstan 420008, Russia; ^3^Institute of Physics, Kazan Federal University, Kremlevskaya St. 16, Kazan 420008, Russia

## Abstract

Cadmium (Cd) and lead (Pb) are heavy metals, important environmental pollutants, and potent toxicants to organism. Lactic acid bacteria (LAB) have been reported to remove Cd and Pb from solutions and therefore represent a useful tool for decontamination of food and beverages from heavy metals. Heavy metal ion binding by LAB was reported as metabolism-independent surface process. In this work ten* Lactobacillus* strains were investigated with respect to hydrophobicity, Lewis acid-base, and electrostatic properties of their outer cell surface in order to characterize their Cd and Pb removal capacity. Seven* L. plantarum* and* L. fermentum* strains were shown to remove Cd from culture medium. The metabolism-dependent accumulation mechanism of Cd removal was proposed based on extended character of Cd binding and lack of correlation between any of the surface characteristics and Cd removal. The results of this study should be considered when selecting probiotic strains for people at risk of Cd exposure.

## 1. Introduction

Lead (Pb) and cadmium (Cd) are the two most abundant toxic heavy metals in the environment, reported in the Priority List of Hazardous Substances on the 2nd and 7th places, respectively [1]. They are biologically nonessential and nondegradable and tend to accumulate in exposed organisms. Pb exposure induces neurologic and hematological dysfunctions, cardiovascular, hepatic, and renal damage, and reproductive disorders in the human body. It is particularly harmful to the young children [2]. Cd toxicity is associated primarily with renal, skeletal, and pulmonary dysfunctions [3]; hepatic, reproductive, and cardiovascular disorders are also described [4]. Besides, International Agency for Research on Cancer (IARC) classifies Cd as a group I human carcinogen.

Despite the constitutive efforts to protect the health of children and adults from hazardous heavy metals, occupational and environmental exposures to Pb and Cd remain a serious problem in many countries. Lactic acid bacteria (LAB) and more particularly lactobacilli were reported to bind heavy metals and thus represent a promising approach for decontamination of heavy metals in food and water and perhaps gastrointestinal tract as well, extending areas of LAB applications in food industry and probiotics. In contrast to conventional physicochemical techniques microbial metal ion binding exhibits fine specificity and is environmentally friendly, of low-cost, and efficient at low metal ion concentrations [5]. To date, the ability to bind Cd and Pb was reported for several probiotic and food-grade* Lactobacillus* strains:* L. rhamnosus *GG [5–7], LC705 [6–8],* L. johnsonii* Lj1 [6],* L. casei* Shirota [5, 6], and* L. fermentum* ME3 [5, 9]. In addition,* L. amylovorus, L. reuteri*, and* L. dextrinicus *strains, isolated from Cd- and Pb-contaminated mud and sludge samples, were recognized as Cd- and Pb-removing probiotic strains [10].

Although social benefit from LAB in bioremediation of contaminated food and humans themselves is well recognized, detoxication mechanisms of lactobacilli are still controversial. Heavy metal binding was reported to be strain, temperature, and pH dependent and happened efficiently at low concentration ranges commonly observed in foods [6, 8]. Due to rapid uptake of Cd and Pb from aqueous solution, the mechanism of passive binding of metal ions to the surface of bacteria was suggested rather than accumulation inside the cell [5]. According to [9], several reversible mechanisms such as ion exchange and precipitation are involved in Cd and Pb binding by LAB. Reduction of Cd and Pb removal under low pH [5] and in the presence of other cations [9] supports the idea that metal uptake is determined by physical adsorption. Using electron microscopy and Fourier transform infrared (FTIR) spectroscopy in two* Lactobacillus kefir *strains, CIDCA 8348 and JCM 5818, it was shown that S-layer proteins interact with Cd and Pb and adjust their structure to the presence of the metal ions [11]. Yet, the absence of correlation between cell charge and removal of Cd and Pb questions the involvement of electrostatic interactions between heavy metals and LAB [7]. Besides, no data were reported so far about the possibility of accumulation of heavy metals inside* Lactobacillus* cells.

The aim of this work was to determine the cell surface characteristics and the potential ability to remove Cd and Pb from aqueous solutions and culture medium with ten* Lactobacillus *strains, including four* L. plantarum *strains, three* L. fermentum *strains,* L. brevis*,* L. buchneri*, and* L. rhamnosus*. Some of these strains were specifically isolated from probiotics, dairy products, and silage. Investigations of hydrophobic/hydrophilic character and Lewis acid-base interactions were performed by using the microbial adhesion to solvents (MATS) method, and electrostatic properties were studied by microelectrophoresis.

## 2. Materials and Methods

### 2.1. Heavy Metals

Cd (2 mg/mL) and Pb (2 mg/mL) stock solutions in Milli-Q water were prepared from Cd(NO_3_)_2_ and Pb(NO_3_)_2_ (Sigma-Aldrich), respectively. Metal solutions were added to the bacterial culture medium after autoclaving and cooling at c. 40°C.

### 2.2. Bacterial Strains

The following* Lactobacillus *strains were used in this study:* Lactobacillus plantarum* 8PA3 (“Lactobacterin dry”, Biomed, Russia),* Lactobacillus plantarum* B-578 (All-Russian Collection of Microorganisms, VKM),* Lactobacillus plantarum* S1 (Silage, Chistopolsky region, Tatarstan Rep., Russia),* Lactobacillus plantarum* Ga (“Gastropharm,” Biovet, Bulgaria),* Lactobacillus fermentum* Na (“Narine,” Narex, Armenia),* Lactobacillus fermentum *3-2 (sour-milk drink “Ayran,” FoodMilk),* Lactobacillus fermentum* 3-3 (sour-milk drink “Dar Gor,” FoodMilk),* Lactobacillus brevis* DSM-20054,* Lactobacillus buchneri* DSM-20057 (German Collection of Microorganisms and Cell Cultures, DSMZ), and* Lactobacillus rhamnosus* I2L (Russian National Collection of Industrial Microorganisms, VKPM). Isolation and identification of bacteria are described elsewhere [12].

### 2.3. Growth Media and Culture Conditions

Lactobacilli were cultured in de Man, Rogosa, Sharpe (MRS) broth (BD Difco) under microaerophilic conditions at 37°C.

Toxicity of heavy metals was studied using a Tecan Infinite F200 PRO (Switzerland) microplate reader. Overnight cultures of the lactobacilli were diluted 1 : 50 with fresh MRS broth containing 0–50 mg/L Pb or Cd and loaded into sterile polystyrene 96-well microplates (flat bottom, CellStar Greiner Bio-One). Microplates were incubated at 37°C and measurements of the optical density at 600 nm (OD_600_) were automatically recorded each 30 min with 20 s shaking cycles before measurements were started. Growth analysis of the lactobacilli cultures in the presence of Pb or Cd was performed along with controls without heavy metals to obtain reference growth curves, as well as with sterile media controls as background readings.

The bacteria for characterization of surface properties were cultured in MRS broth for 18–20 h, harvested by centrifugation at 5000 rpm for 10 min, and washed three times with appropriate KNO_3_ solution.

The supernatant of the bacteria grown for 24 h in MRS broth with 5 mg/L Pb or Cd was used for measurements of Cd and Pb concentrations with atomic absorption spectrometry.

### 2.4. Surface Characterization of Bacteria

#### 2.4.1. MATS Method

Microbial adhesion to solvents (MATS) was measured according to the method of Rosenberg et al. [13] with some modifications of Bellon-Fontaine et al. [14]. Bacteria were harvested in the stationary phase by centrifugation at 5000 rpm for 10 min, washed three times, and resuspended to an optical density of 0.4 at 400 nm (*A*_0_) in 0.1 M KNO_3_ (pH 6.2). 0.2 mL of solvent was added to 1.2 mL of cell suspension. After a 10 min preincubation at room temperature, the two-phase system was mixed on a vortex for 2 min and incubated for 15 min for phase separations. The aqueous phase was gently taken out to measure its optical density at 400 nm (*A*_1_). The percentage of microbial adhesion to solvent was calculated as (1 − *A*_1_/*A*_0_) × 100.

Three different solvents were tested in this study: n-hexadecane (Sigma-Aldrich), which is an apolar solvent; chloroform (Sigma-Aldrich), a monopolar and acidic solvent; and ethyl acetate (Sigma-Aldrich), a monopolar and basic solvent. Bacterial adhesion to n-hexadecane reflects cell surface hydrophobicity or hydrophilicity. The values of MATS obtained with the two other solvents, chloroform and ethyl acetate, were regarded as a measure of electron donor (basic) and electron acceptor (acidic) characteristics of bacteria, respectively [14].

#### 2.4.2. Zeta Potential

Zeta potential (*ζ*) was measured to determine the cell surface net charge of the bacteria. A Zetasizer Nano ZS (Malvern Instruments, UK) was used to measure the electrophoretic mobility, with conversion to zeta potential using Smoluchowski's approximation. Measurements were performed with cells suspended in 1 mM KNO_3_ (рН = 6.0). The samples were placed into standard U-shaped zeta/size cells and measured in triplicate at 25°C

The influence of Cd and Pb on the bacterial surface charge was also investigated. Prior to the measurements, cells were incubated for 1 h at 180 rpm in 1 mM KNO_3_ (рН = 6.0) spiked with 5 mg/L Pb or Cd. After that, bacteria were separated by centrifugation at 5000 rpm for 5 min and resuspended in 1 mM KNO_3_ (рН = 6.0).

#### 2.4.3. Measurement of Cadmium and Lead

Pb and Cd concentrations in the supernatants were measured using atomic absorption spectrometer MGA-915 MD (Lumex, Russia) with graphite tube atomizer and autosampler. Standard pyrocoated graphite furnaces (length 28 mm, internal diameter 6 mm) with longitudinal heating (PerkinElmer, USA) and high pure argon for inert atmosphere were applied. The spectrometer was equipped with special accessory for two-stage probe atomization Atzond-1 (Atzond, Russia). Samples of MRS broth spiked with 5 mg/L Pb or Cd were used as quality control samples. Metal removal rate was expressed in percentage. Changes of metal concentration in the samples over 3% (Pb) and 7% (Cd) can be clearly distinguished at *P* = 0.7.

### 2.5. Statistical Analysis

All experiments were independently conducted two or three times, and each assay was performed in triplicate. The results were expressed as the means ± standard deviation. Student's *t*-test (for paired or unpaired samples) was used to compare the results; the differences were considered significant when *P* < 0.05. For correlations, the Pearson correlation coefficient (*r*) was used.

## 3. Results

### 3.1. Toxicity of Cd and Pb towards* Lactobacillus*

**Figure 1:**
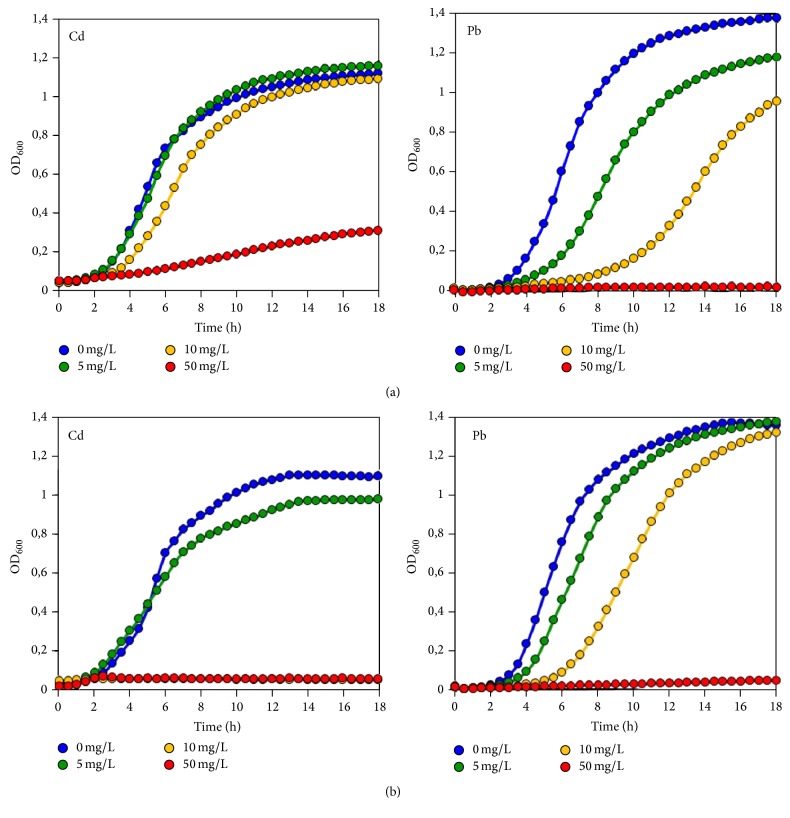
Growth of* Lactobacillus plantarum* S1 (a) and* L. buchneri* 20057 (b) in the presence of Cd or Pb (see growth curves of all* Lactobacillus* strains in Supplemental Figures S1–S3).

The lowest tested concentration of Cd (5 mg/L) showed no influence on the growth of lactobacilli except* L. fermentum* 3-2, in which presence of 5 mg/L Cd led to decrease of maximum optical density values (see Supplemental Figure S2(b) available online at https://doi.org/10.1155/2017/9869145). Furthermore, in* L. plantarum *8PA3 and* L. fermentum*, 3-2 cultures with 5 mg/L Cd slopes of the growth curves during the exponential phase were slightly higher than those in Cd-free controls (Supplemental Figures S1(a) and S2(a)). Application of 10 mg/L Cd resulted in reduced OD_600_ values in the stationary phase of* L. plantarum *8PA3,* L. plantarum *В-578, and* L. fermentum *3-2 cultures and totally inhibited growth of* L. brevis *20054,* L. buchneri *20057, and* L. rhamnosus *I2L. Cd in the highest tested concentration (50 mg/L) was toxic for all tested lactobacilli. Specifically, cultures of* L. plantarum *and* L. fermentum* exhibited significant reduction in growth rate and cultures of* L. brevis *20054,* L. buchneri *20057, and* L. rhamnosus *I2L did not show bacterial growth within 18 h (Figure 1, Supplemental Figures S1–S3).

In all tested cultures addition of Pb at concentrations 5 and 10 mg/L resulted in a shallower growth curve slope and extended lag phase compared with Pb-free controls. Furthermore, despite the delayed onset of growth and lower growth rate, the addition of Pb at these concentrations did not lead to lower maximum optical density values in lactobacilli cultures except* L. plantarum *S1, in which Pb led to decrease of OD_600_ on 14.5% and 30.4% at 5 and 10 mg/L Pb, correspondingly (Figure 1(a)). Concentration 50 mg/L Pb revealed complete growth inhibition in all lactobacilli cultures (Figure 1, Supplemental Figures S1–S3).

### 3.2. Physicochemical Properties of Bacterial Cell Surface

The adhesive characteristics of* Lactobacillus *strains to n-hexadecane, chloroform, and ethyl acetate are shown in Table 1. The results indicated that most strains were fully hydrophilic, since very low percentages of bacteria (6.6–28.7%) adhered to n-hexadecane, an apolar solvent. The most hydrophobic strains were* L. plantarum *B-578 (52.0 ± 6.4%),* L. brevis *20054 (63.1 ± 5.6%), and* L. buchneri *20057 (66.9 ± 6.3%).

Bacterial adhesion to chloroform and ethyl acetate was tested to assess the Lewis acid-base characteristics of the bacterial cell surfaces. The affinities with chloroform, which is an acidic solvent and electron acceptor, varied significantly between tested strains. The least affinity with chloroform was observed in* L. fermentum* Na (9.8 ± 1.2%), while the greatest affinities were observed in* L. plantarum *B-578 (88.8 ± 3.6%),* L. fermentum *3-2 (93.8 ± 2.2%),* L. brevis *20054 (94.6 ± 0.1%), and* L. buchneri *20057 (97.1 ± 0.1%). Adhesion to ethyl acetate, which is a basic solvent and electron donor, was low with all bacteria studied, ranging from 14.6 ± 2.8% to 35.6 ± 3.1%.

The cell surface net charge of the bacteria was examined by microelectrophoresis, which measures zeta potentials of microorganisms in the stationary phase. KNO_3_ solution was used as reference medium to avoid nonspecific absorption of ions on cell surfaces [15]. In general, the net surface charge of the studied strains was negative, ranging from −34.9 ± 6.8 mV (*L. plantarum *S1) to −7.4 ± 0.9 mV (*L. fermentum* 3-3) (Table 1). Furthermore, zeta potentials differed significantly both between species and strains.

### 3.3. Binding of Cd and Pb by* Lactobacillus *Cells

First, the potential of* Lactobacillus* cells to bind heavy metals was studied as the difference in cell surface net charge after incubation with Cd or Pb. A contact time of 1 h was chosen according to Halttunen et al. [5]. In all* Lactobacillus* strains after 1 h incubation in aqueous solutions containing 10 mg/L of Cd or Pb, differences towards more positive zeta potentials were not observed (not presented). Conversely, cell surface electronegativity decreased after incubation with Cd and Pb, but reductions were not statistically significant.

The removal of Cd and Pb from MRS broth is presented in Table 2.* Lactobacillus *cells did not remove Pb from MRS broth, nor did* L. brevis *20054,* L. buchneri *20057, and* L. rhamnosus *I2L with Cd.* L. plantarum *and* L. fermentum *strains demonstrated removal of Cd, ranging from 8 to 16%. The most efficient removal of Cd was observed with* L. plantarum *В-578 (16%). However, no correlation between any of the surface characteristics and removal of Cd was observed.

## 4. Discussion

The results demonstrate that* Lactobacillus* strains were highly tolerant to Cd and Pb.* L. brevis *20054,* L. buchneri *20057, and* L. rhamnosus *I2L were most sensitive to heavy metals because they demonstrated considerable growth reduction at 10 and 50 mg/L of Cd whereas* L. fermentum *and* L. plantarum* strains continued to grow at these concentrations. Bhakta et al. [10] showed that Pb and Cd resistant* Lactobacillus *strains more likely demonstrated increased Pb and Cd removal efficiency.* L. fermentum *and* L. plantarum* strains are therefore considered to be potential Pb and Cd removing bacteria.

There are two basic mechanisms of metal ion binding by microorganisms: bioaccumulation—metabolism associated process in which metal ions penetrate plasma membrane and accumulate inside the cell—and biosorption—the metabolism-independent binding of metal ions to the cell surface [16]. Mechanisms such as adsorption, ion exchange, complexation, chelation, and microprecipitation have been proposed to be involved in metal biosorption [17]. Since metal ion binding is a surface process we assessed physicochemical properties of* Lactobacillus* cells and their impact on binding of Pb and Cd.

First, cell surface hydrophobicity was examined by measuring microbial adhesion to n-hexadecane in a two-phase system. The results indicated that most microorganisms studied were relatively hydrophilic (Table 1). The hydrophilic nature of lactobacilli has often been encountered in previous studies [15, 18, 19]. Three strains,* L. plantarum *B-578 (52.0 ± 6.4%),* L. brevis *20054 (63.1 ± 5.6%), and* L. buchneri *20057 (66.9 ± 6.3%), were hydrophobic. The hydrophobic/hydrophilic properties resulted from proteins and polysaccharides on the bacterial cell surface. The presence of proteinaceous compounds at the cell surface results in a higher hydrophobicity [20–22], whereas a hydrophilic surface is associated with the presence of polysaccharides [22–24]. (Lipo)teichoic acids, which are hydrophobic, might have an effect on hydrophobicity as well, but it is unclear [22, 25]. It is well known that lactobacilli show great diversity in cell surface structure and composition and are able to modify their surface properties in response to environmental changes [26]. In all probabilities, species and strain specific variations in the cell surface hydrophobicity result from different expression of certain surface components (adhesins, polysaccharides, and proteins).

Further chloroform and ethyl acetate were used to assess the electron donor (basic) and electron acceptor (acidic) characteristics of bacterial surface, respectively (Table 1), which are attributed to Lewis acid-base interactions [14, 15]. The bacterial affinities to ethyl acetate were low in all strains tested indicating the nonacidic and poor electron acceptor properties of lactobacilli. The high affinities to chloroform in* L. plantarum *B-578 (88.8 ± 3.6%),* L. fermentum *3-2 (93.8 ± 2.2%),* L. brevis *20054 (94.6 ± 0.1%), and* L. buchneri *20057 (97.1 ± 0.1%) indicate the basic (electron donor) character of the bacterial cell, which is probably related to the presence of a carboxylic (-COO^−^) and hydrogen sulfite (-HSO_3_^−^) groups on the microbial surface [14, 15].

We subsequently studied electrostatic cell surface properties of lactobacilli by measuring the electrophoretic mobility in microelectrophoresis, which is a common method to determine cell surface charge. All* Lactobacillus* strains displayed an overall electronegative charge, which differed between species and strains from −7.4 ± 0.9 to −34.9 ± 6.8 mV, similar to zeta potential profiles that were previously reported for other lactobacilli [15, 18, 22, 24, 25]. Such profiles indicate that the surface of the cells was to large extent dominated by anionic compounds, such as phosphate groups, involved in (lipo)teichoic acids, and carboxylate groups of acidic polysaccharides and proteins [22, 23]. Strikingly, no relationships seemed to exist between the electrostatic properties and the electron donor or electron acceptor profile of the microorganisms (*r* = 0.17 and *r* = 0.24, resp.). This result is consistent with earlier data of Pelletier et al. [15].

Several reports indicate involvement of electrostatic interactions in bacterial binding of heavy metals. This notice is supported by pH dependent manner of Cd and Pb binding by* Bacillus subtilis* [27],* Pseudomonas putida* [28],* Citrobacter *sp. [29],* L. rhamnosus* GG,* L. fermentum* ME3,* B. lactis* Bb12, and* B. longum* 46 [5]. Reduced metal binding at lower pH may result from competition for negatively charged binding sites between heavy metal cations and protons (H^+^) [30]. Similarly, the presence of other cations reduced Cd and Pb binding with* L. fermentum* ME3 and* B. longum* 46, probably, due to competition between these metals for the binding sites [9]. Involvement of anionic surface groups in metal binding with* L. fermentum* ME3,* B. longum* 46, and isolated* B. subtilis* cell walls was also verified by reduction of cation uptake after inactivation of phosphoryl and carboxyl groups [9, 31, 32].

Our results of physicochemical properties indicated electronegative nonacidic character of all lactobacilli tested. Four strains, namely,* L. plantarum *B-578,* L. fermentum *3-2,* L. brevis *20054, and* L. buchneri *20057, possessed strong electron donor properties and were therefore considered to have the larger potential for metal binding among tested lactobacilli. According to zeta potential profile,* L. plantarum *S1, which showed the most negative surface charge, has the strongest potential for metal binding. Yet, its high electronegativity is not confirmed by weak electron donor properties. The surface of* Lactobacillus *cells, like other Gram-positive bacteria, is composed of a thick peptidoglycan layer, (lipo)teichoic acids, polysaccharides, and proteins, including S-layer (glycol)proteins [26]. These structures contain different kinds of charged groups like carboxyl, hydroxyl, and phosphate groups. Lactobacilli have therefore a great number of different possible ligands capable of binding cationic ions like Pb and Cd.

To test Cd and Pb binding by lactobacilli we employed two different approaches. First, the differences of cell charge were measured after 1 h contact with heavy metals, but no shift towards more positive zeta potentials was detected. These results indicate that lactobacilli did not bind Cd and Pb ions at these conditions. Next, we studied the decrease of Cd and Pb concentrations in MRS broth after 24 h growth and showed that* L. plantarum *and* L. fermentum *strains removed Cd from culture medium (Table 2). Yet, this removal was much lower compared to that previously reported for* L. fermentum* ME3 [5, 9].

No relationship between heavy metal removal and tolerance was indicated. It should be noted that three strains,* L. brevis *20054,* L. buchneri *20057, and* L. rhamnosus *I2L, which demonstrated the strongest sensitiveness to Cd, were not able to bind it. Meanwhile,* L. fermentum *Na, tolerant to all Cd concentrations studied, did not remove its ions from culture medium. Earlier the absence of clear relationship between the resistant patterns and metal removal efficiencies was reported for other LAB, thus indicating the existence of variations in resistant mechanism among the LAB [10].

In agreement with other studies of Halttunen et al. [7], no correlation between any of the surface characteristics (hydrophobicity, Lewis acid-base properties, and surface charge) and removal of Cd and Pb was observed. These paradoxical data may result from metabolism-dependent accumulation of Cd inside the bacteria, which does not depend much on physical properties of cell surface. Extended character of Cd removal by tested lactobacilli, which occurred during 24 h incubation, but not within 1 h, favors accumulation mechanism of metal ion binding, rather than biosorption. Studies on Pb and Cd are often conducted together, as the elements seem to react with bacterial species in similar ways. Yet, we did not observe removal of Pb with all tested* Lactobacillus* strains, while* L. plantarum *and* L. fermentum *strains removed Cd from culture medium. Perhaps, this difference results from larger ionic radius of Pb if compared with Cd.

According to Monachese et al. [33], bioaccumulation and biosorption of heavy metals by LAB both are prosperous detoxification strategies as they prevent the exposure of heavy metals to body cells and tissues. Our current work expands knowledge about cell surface of lactobacilli and reveals Cd decontamination potential of four* L. plantarum* and three* L. fermentum* strains, six with known probiotic properties and one firstly isolated from silage. Continued investigation should provide deeper understanding of mechanisms underpinning Cd removal and application of the microorganisms in dietary strategies for people at risk of Cd exposure.

## Supplementary Material

Growth curves of Lactobacillus strains incubated in MRS broth supplemented with 0-50 mg/L Pb or Cd.
Supplemental figure S1. Growth of Lactobacillus plantarum 8PA3 (a), L. plantarum B-578 (b), L. plantarum S1 (c), L. plantarum Ga (d) in the presence of Cd or Pb.
Supplemental figure S2. Growth of Lactobacillus fermentum Na (a), L. fermentum 3-2 (b), L. fermentum 3-3 (c) in the presence of Cd or Pb.
Supplemental figure S3. Growth of Lactobacillus brevis 20054 (a), L. buchneri 20057 (b), L. rhamnosus I2L (c) in the presence of Cd or Pb.

## Figures and Tables

**Table 1:** Physicochemical properties of bacterial cell surface.

Species and strains	% of adhesion (±SD)^a^ to			*ζ*-potential
	Hexadecane	Ethyl acetate	Chloroform	
*L. plantarum*				
8PA3	9.3 ± 2.2	34.5 ± 3.0	17.9 ± 1.6	−24.8 ± 2.5
B-578	52.0 ± 6.4	19.1 ± 2.0	88.8 ± 3.6	−27.6 ± 1.9
S1	6.6 ± 1.6	15.1 ± 2.3	29.3 ± 3.4	−34.9 ± 2.3
Ga	17.6 ± 3.8	14.6 ± 2.8	29.9 ± 2.6	−12.1 ± 1.2
*L. fermentum*				
Na	20.8 ± 3.1	18.4 ± 2.7	9.8 ± 1.2	−11.3 ± 0.7
3-2	27.9 ± 3.3	23.8 ± 2.2	93.8 ± 2.2	−19.3 ± 1.8
3-3	8.7 ± 1.8	10.7 ± 1.6	20.1 ± 2.1	−7.4 ± 0.9
*L. brevis* 20054	63.1 ± 5.6	26.5 ± 2.0	94.6 ± 0.1	−14.8 ± 1.8
*L. buchneri* 20057	66.9 ± 6.3	35.6 ± 3.1	97.1 ± 0.1	−20.7 ± 1.7
*L. rhamnosus* I2L	28.7 ± 3.3	21.3 ± 2.7	34.8 ± 4.0	−21.5 ± 2.2

^a^Means ± standard deviations of two measures of three separate experiments.

**Table 2:** Removal of Cd from MRS broth by *Lactobacillus* strains.

Species and strains	% Cd removed
*L. plantarum*	
8PA3	8
B-578	16
S1	8
Ga	8
*L. fermentum*	
Na	4
3-2	8
3-3	12
*L. brevis* 20054	0
*L. buchneri* 20057	0
*L. rhamnosus* I2L	0

The bacteria were incubated 24 h in MRS broth supplemented with 5 mg/L Cd.
