# Supplementary material for: Assessment of Resistance and Bioremediation Ability of Lactobacillus Strains to Lead and Cadmium
Source: Int J Microbiol. 2017 Jan 4;2017:9869145. doi: 10.1155/2017/9869145 (PMC5241453; doi:10.1155/2017/9869145)
Supplement: Supplementary file 1 — Growth curves of Lactobacillus strains incubated in MRS broth supplemented with 0-50 mg/L Pb or Cd. Supplemental figure S1. Growth of Lactobacillus plantarum 8PA3 (a), L. plantarum B-578 (b), L. plantarum S1 (c), L. plantarum Ga (d) in the presence of Cd or Pb. Supplemental figure S2. Growth of Lactobacillus fermentum Na (a), L. fermentum 3-2 (b), L. fermentum 3-3 (c) in the presence of Cd or Pb. Supplemental figure S3. Growth of Lactobacillus brevis 20054 (a), L. buchneri 20057 (b), L. rhamnosus I2L (c) in the presence of Cd or Pb. [file 9869145.f1.pdf]

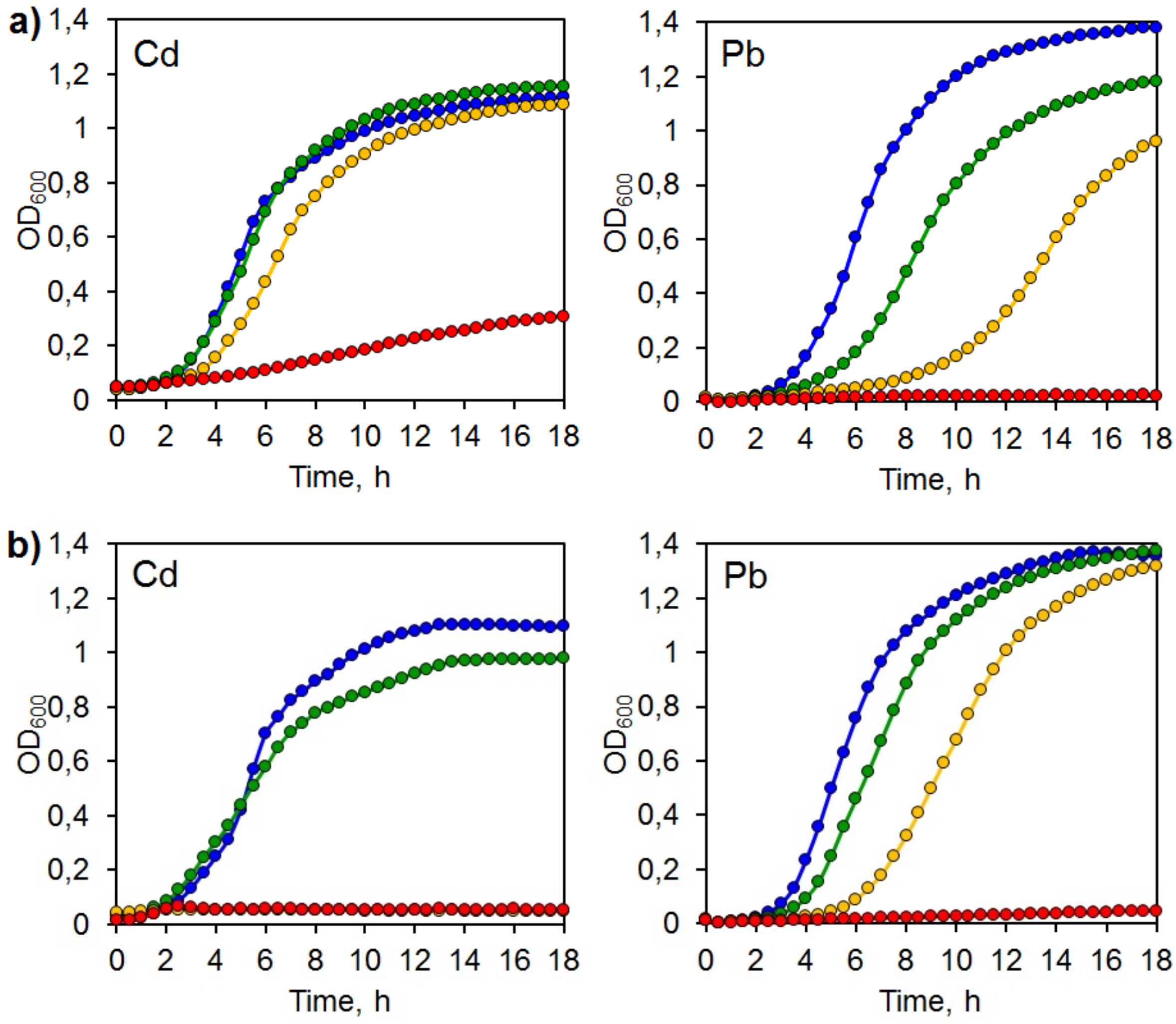

a)
b)
Cd
Pb
OD$_{600}$
Time, h
1,4
1,2
1
0,8
0,6
0,4
0,2
0
0 2 4 6 8 10 12 14 16 18

Supplemental data

Fig. S1. Growth of *Lactobacillus plantarum* 8PA3 (a), *Lactobacillus plantarum* B-578 (b), *Lactobacillus plantarum* S1 (c), *Lactobacillus plantarum* Ga (d) in the presence of Cd or Pb. ( ) 0 mg/L; ( ) 5 mg/L; ( ) 10 mg/L; ( ) 50 mg/L.

Fig. S2. Growth of *L. fermentum* Na (a), *L. fermentum* 3-2 (b), *L. fermentum* 3-3 (c) in the presence of Cd or Pb. ( ) 0 mg/L; ( ) 5 mg/L; ( ) 10 mg/L; ( ) 50 mg/L.

Fig. S3. Growth of *L. brevis* 20054 (a), *L. buchneri* 20057 (b), *L. rhamnosus* I2L (c) in the presence of Cd or Pb. ( ) 0 mg/L; ( ) 5 mg/L; ( ) 10 mg/L; ( ) 50 mg/L.

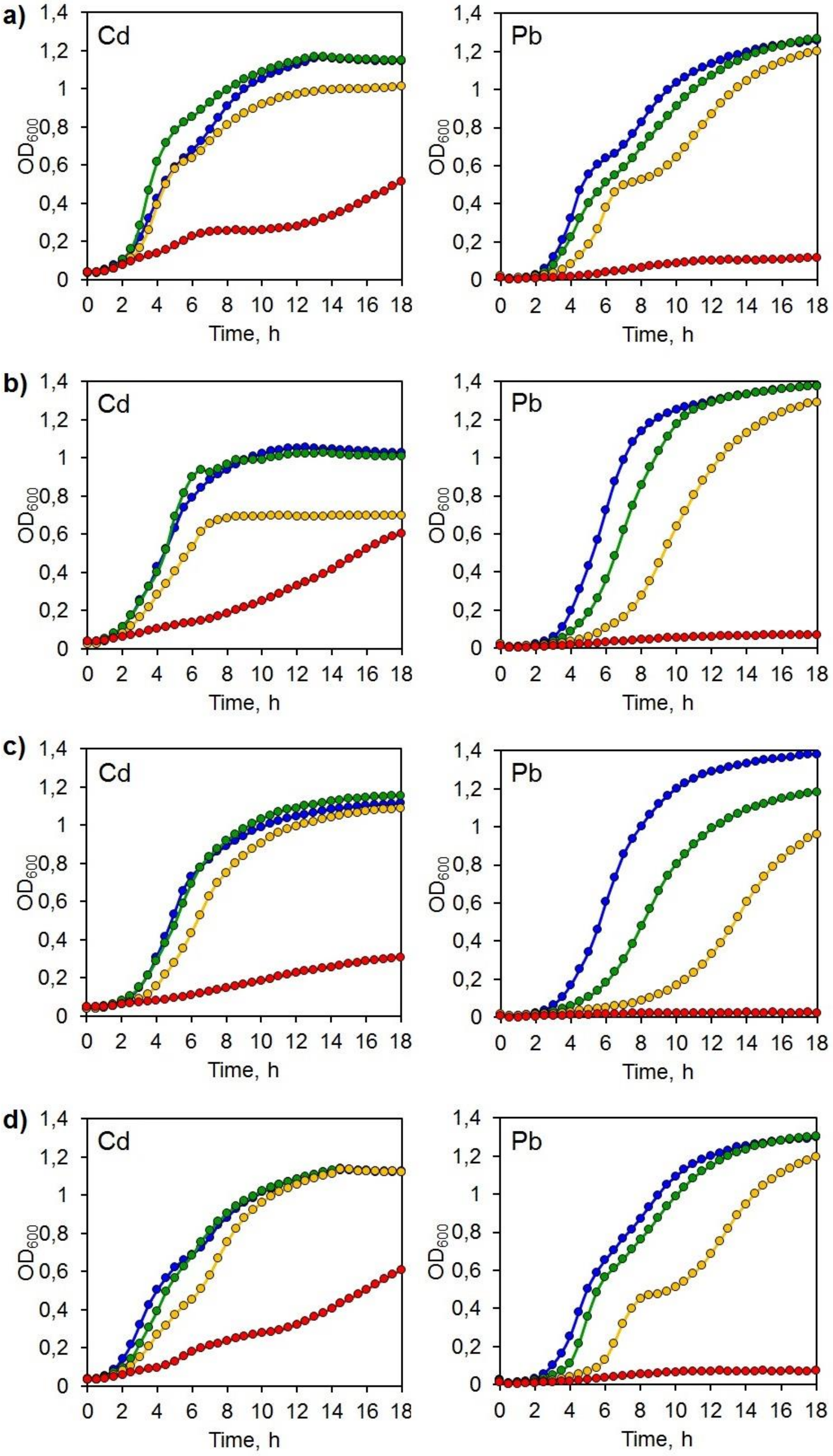
a)
b)
c)
d)
Cd
Pb
OD600
Time, h
0
0,2
0,4
0,6
0,8
1
1,2
1,4
0 2 4 6 8 10 12 14 16 18

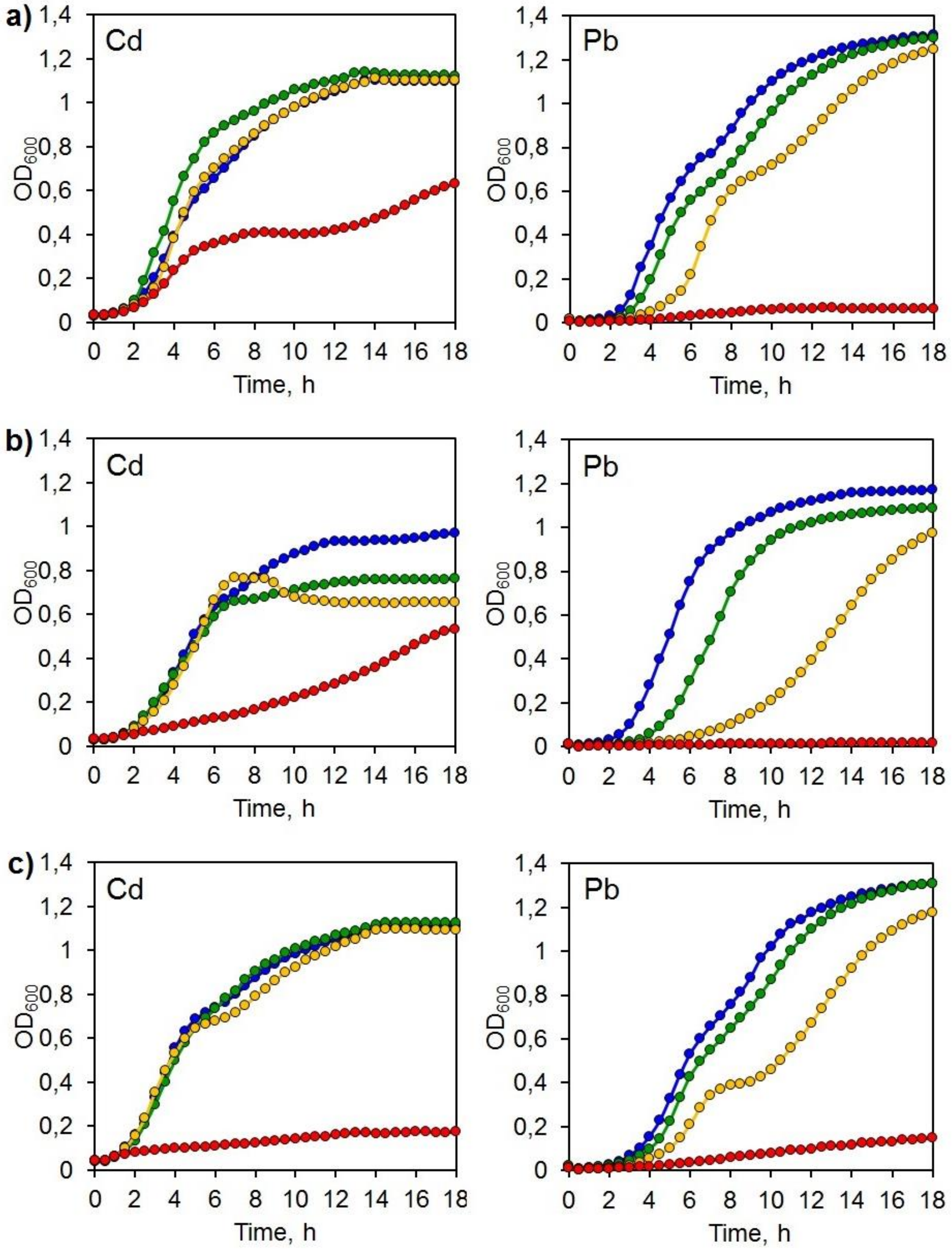

a)
Cd
Pb
b)
Cd
Pb
c)
Cd
Pb
$OD_{600}$
Time, h

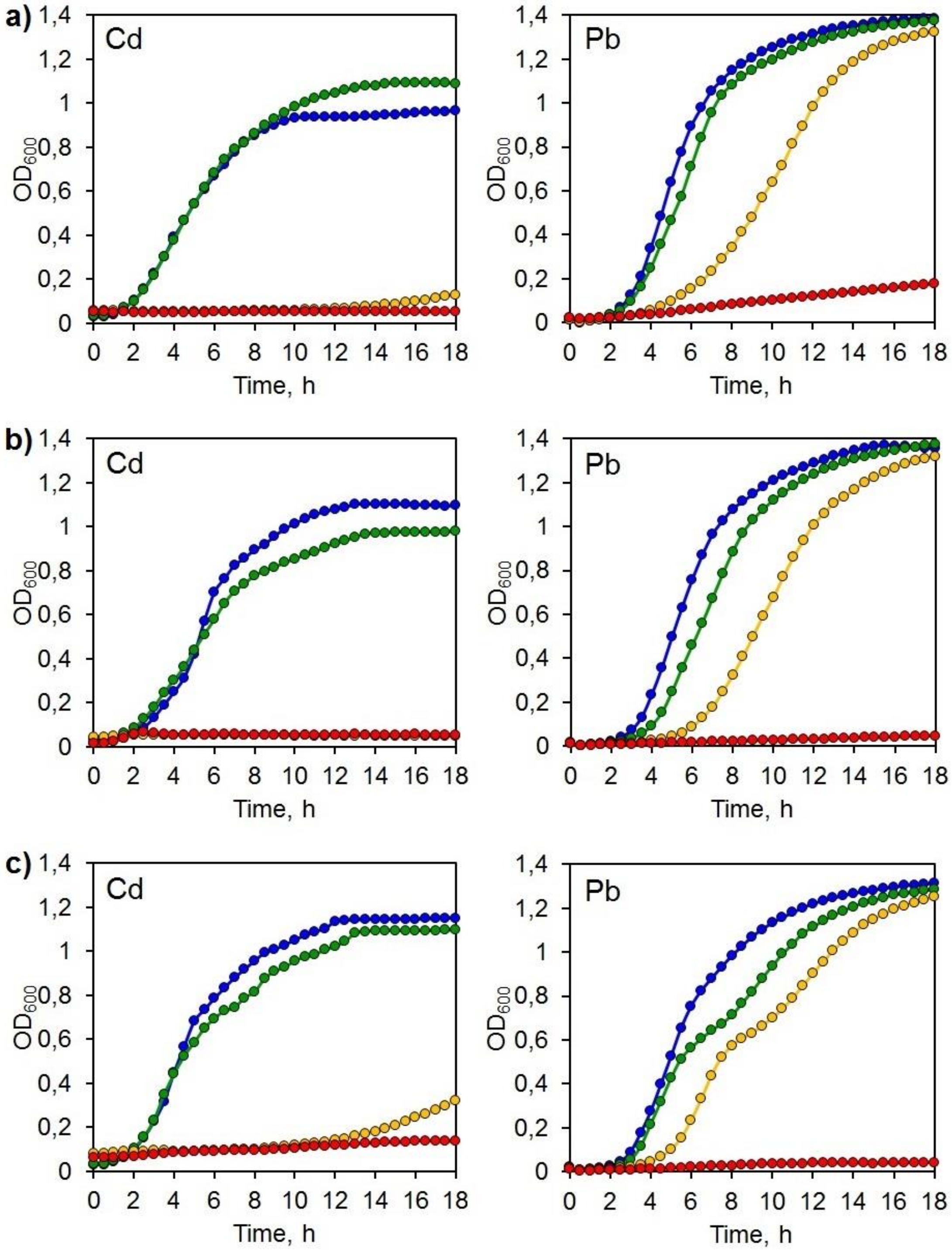

a)
Cd
Pb
b)
Cd
Pb
c)
Cd
Pb
OD600
Time, h
1,4
1,2
1
0,8
0,6
0,4
0,2
0
0 2 4 6 8 10 12 14 16 18
